# Mechanics of the human foot during walking on different slopes

**DOI:** 10.1371/journal.pone.0286521

**Published:** 2023-09-11

**Authors:** Nikolaos Papachatzis, Kota Z. Takahashi

**Affiliations:** 1 Department of Mechanical Engineering & Materials Science, Yale University, New Haven, Connecticut, United States of America; 2 Department of Biomechanics, University of Nebraska at Omaha, Omaha, Nebraska, United States of America; 3 Department of Health & Kinesiology, University of Utah, Salt Lake City, Utah, United States of America; Brunel University London, UNITED KINGDOM

## Abstract

When humans walk on slopes, the ankle, knee, and hip joints modulate their mechanical work to accommodate the mechanical demands. Yet, it is unclear if the foot modulates its work output during uphill and downhill walking. Therefore, we quantified the mechanical work performed by the foot and its subsections of twelve adults walked on five randomized slopes (−10°, −5°, 0°, +5°, +10°). We estimated the work of distal-to-hindfoot and distal-to-forefoot structures using unified deformable segment analysis and the work of the midtarsal, ankle, knee, and hip joints using a six-degree-of-freedom model. Further, using a geometric model, we estimated the length of the plantar structures crossing the longitudinal arch while accounting for the first metatarsophalangeal wrapping length. We hypothesized that compared to level walking, downhill walking would increase negative and net-negative work magnitude, particularly at the early stance phase, and uphill walking would increase the positive work, particularly at the mid-to-late stance phase. We found that downhill walking increased the magnitude of the foot’s negative and net-negative work, especially during early stance, highlighting its capacity to absorb impacts when locomotion demands excessive energy dissipation. Notably, the foot maintained its net dissipative behavior between slopes; however, the ankle, knee, and hip shifted from net energy dissipation to net energy generation when changing from downhill to uphill. Such results indicate that humans rely more on joints proximal to the foot to modulate the body’s total mechanical energy. Uphill walking increased midtarsal’s positive and distal-to-forefoot negative work in near-equal amounts. That coincided with the prolonged lengthening and delayed shortening of the plantar structures, resembling a spring-like function that possibly assists the energetic demands of locomotion during mid-to-late stance. These results broaden our understanding of the foot’s mechanical function relative to the leg’s joints and could inspire the design of wearable assistive devices that improve walking capacity.

## Introduction

Quantifying how the mechanical demands of different locomotor gaits alter the leg’s mechanical work profile has helped us identify how various legged animals utilize energy to achieve locomotion [1–8]. When, for example, humans or other bipeds walk or run at a steady speed over a level surface, they use energy conservation mechanisms that allow them to exchange or recycle potential and kinetic energy between steps [9–11]. However, energy is lost during each step of initial foot contact, disrupting the effectiveness with which these mechanisms conserve energy and requiring positive work on the body’s center of mass to maintain the same speed [12]. Therefore, the muscle-tendon structures of the leg’s joints must absorb or dissipate negative work and compensate for the inevitable energy loss (during initial foot contact) by performing positive work. Indeed during locomotion on a level surface for a range of steady-state speeds, certain joints like the ankle do net-positive work (produce energy) [2, 13], while the foot, particularly the heel, performs a large portion of the net negative work (energy dissipation) during early stance [14–17]. Studies have previously explored how joints modulate work and power over a range of steady-state speeds on level or sloped surfaces or/and by changing the body’s net mechanical energy requirements [2, 6, 18–21]. However, the mechanical adaptability of the human foot has been examined primarily in single-stepping tasks [22] or over locomotor tasks on a level surface, like running [14] and walking with added mass [15]. In the present study, we aimed to investigate how the human foot modulates power and work during walking on sloped surfaces that require substantially different net mechanical work on the center of mass to increase (uphill) or decrease (downhill) the body’s potential energy.

For instance, in slope walking at a constant velocity, the vertical mechanical power requirements (i.e., the rate at which work is performed) associated with increasing or decreasing the body’s gravitational potential energy can be calculated as:

Verticalmechanicalpower=m∙g∙sin(θ)∙v

,where *m* is the mass of the body, *g* is gravity, *θ* is the slope in degrees, and *v* is the walking velocity parallel to the slope. As the slope *θ* increases during uphill walking, the net-positive power and work requirements will increase proportionally. In contrast, walking downhill (i.e., negative slope) would increase the net negative power and work requirements. Indeed, when humans and other animals walk or run uphill, the magnitude of the positive work of the leg’s joints, like the ankle and hip, increases [23, 24]. In contrast, when they walk or run downhill, the magnitude of the negative work of these joints increases [23, 25]. Understanding how the human and animal legs utilize energy under different locomotor tasks allowed a deeper understanding of locomotion dynamics [10, 11, 26–29]. However, it is uncertain how the human foot modulates work to accommodate the varying work demands of daily locomotor tasks like walking uphill or downhill.

Consequently, it is reasonable to expect the foot to increase its positive power and work during uphill walking to assist with the increased propulsion demands. On the other hand, during downhill walking, the foot possibly would increase its negative work (i.e., dissipated energy) to decelerate the downward motion of the body. The foot can achieve these various mechanical demands because it comprises anatomical structures that adjust their mechanical function according to the propulsive (i.e., net-positive work) or the dissipative (i.e., net negative work) demands [22, 30, 31]. For instance, active structures like muscles could produce or dissipate energy, and the foot muscles will likely do the same. Riddick et al., (2019) showed that the foot’s intrinsic plantar muscles could generate and dissipate energy during single-stepping tasks, contributing to the net mechanical work on the center of mass [22]. Additionally, the spring-like function of the passive plantar ligaments could contribute to positive work via storing and returning energy. For instance, the elastic function of the plantar fascia that crosses the longitudinal arch is well-documented [32–35]. Previous studies have shown that the dorsiflexion of the first metatarsophalangeal joint (MTPJ) could stretch and enhance the plantar fascia’s elastic function [35–37]. Since walking uphill increases the MTPJ dorsiflexion [38], it may further increase the plantar fascia’s stretch, particularly during mid-stance and, consequently, the energy returned during the late stance phase (i.e., push-off). Moreover, the foot contains structures like the fat pad under the heel that can compress and dissipate negative work during the heel strike and assist with increasing energy dissipation demands of downhill walking. A recent study found during downhill shod walking at a steady speed, the magnitude of negative work done by the foot and shoe complex increases during the early stance phase [39]. That agrees with our recent study, which showed that carrying an additional mass during barefoot walking (1.25 m s^-1^) on a level surface increases the foot’s negative and net negative during the early stance [15]. Hence, walking on slopes can reveal how the human foot adapts its mechanical function to respond to different walking locomotion conditions.

This study aimed to determine how the human foot adapts its mechanical function when walking on uphill and downhill slopes. We used a deformable multi-segment foot model that quantifies the mechanical work production of various subsections of the human foot [15, 40, 41]. We hypothesized that compared to level walking, downhill walking would increase the magnitude of the foot’s negative and net negative work during the entire and during the early stance phase highlighting the foot’s role as a shock absorber. Conversely, we hypothesized that compared to level walking, uphill walking would increase the positive work performed by the foot during the entire stance and particularly at the mid-to-late stance phase. Such mechanical behavior will highlight the foot’s potential to assist propulsion. We further complemented our analysis by estimating the length of the plantar structures crossing the longitudinal arch while accounting for the first metatarsophalangeal (MTPJ) wrapping length [36, 37, 42]. Finally, aiming to qualitatively examine how the foot’s work production compares to more proximal joints, we quantified the total power of ankle, knee, and hip joints using a six-degree-of-freedom model [17, 43]. Determining precisely when and where the foot’s work production is manipulated and how it adapts its mechanical function as a unit to satisfy the various requirements of daily locomotion activities remains challenging. However, improving such knowledge could have clinical implications and improve the design of wearable assistive devices, like exoskeletons [44], foot prostheses [45], and shoes [46].

## Materials and methods

### Participants

Twelve healthy young adults volunteered to participate in this research study (3 females, 9 males; age = 25.5 ± 2.5 yrs; height = 1.74 ± 0.09 m, mass = 75.0 ± 15.5 kg; means ± standard deviation). We determined our sample size based on a power analysis using published data comparing differences in limb mechanical work per step during uphill and downhill walking [3]. For an effect size of 2.98, a sample size of 5 participants will provide 80% power to detect comparable differences, with significance set to α = 0.016. Participants were free of any lower extremity injury, surgery, neurological, musculoskeletal, or pathological condition within the past year. Before participating in the experimental protocol, all participants signed an informed consent form approved by the Institutional Review Board at the University of Nebraska Medical Center.

### Experimental protocol

**Fig 1:**
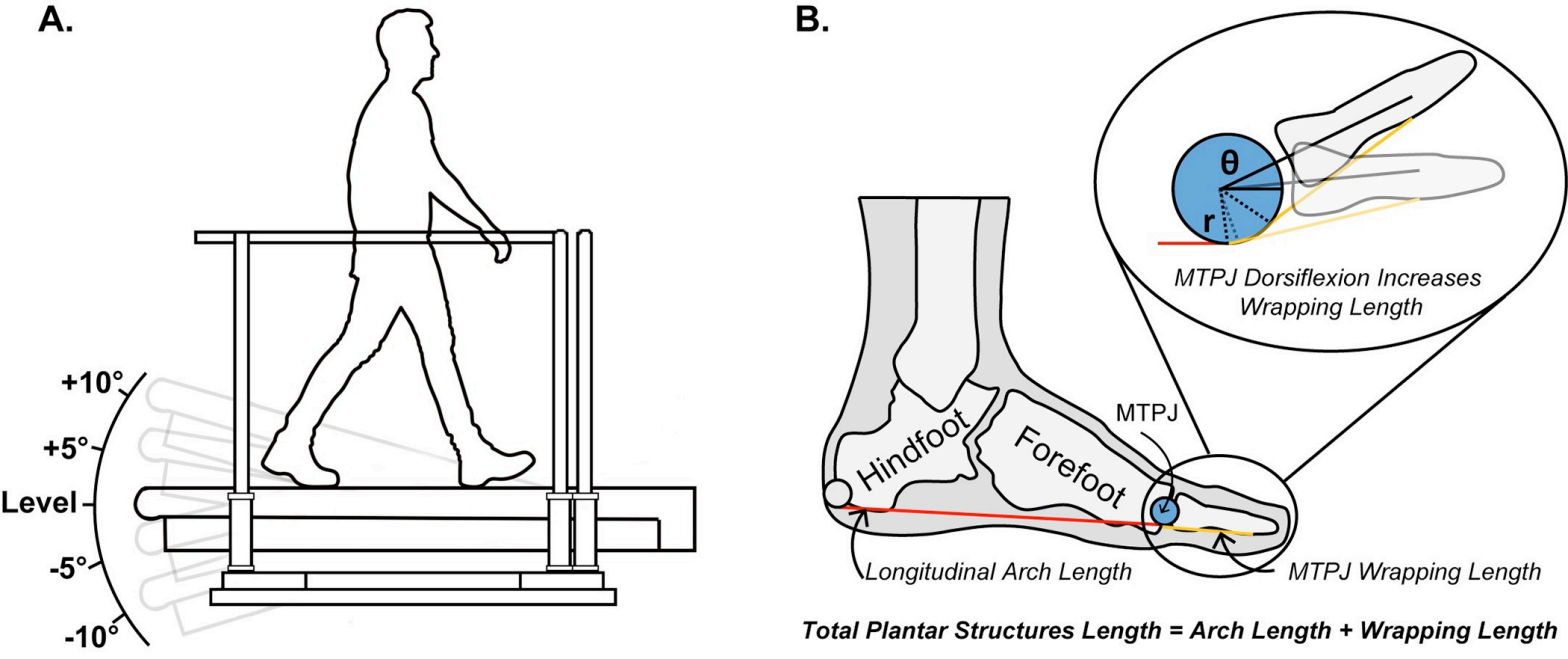
Experimental protocol. **(A)** Participants (*n = 12*) walked barefoot over an instrumented treadmill at 1.25ms^-1^ on five randomized slopes, either level (0°), downhill (−5°, −10°), or uphill (+5°, +10°). We quantified the mechanical power and work of foot sections using methods developed and validated by our previous studies [15, 40, 41]. Additionally, we quantified the total power of the ankle, knee, and hip joints using a six-degree-of-freedom model [17, 43]. **(B)** We approximated the total length of the plantar structures crossing the longitudinal arch while accounting for the metatarsophalangeal (MTPJ) wrapping length. We estimate the horizontal length of the longitudinal arch (i.e., arch length) by measuring the distance (red line) from the hindfoot’s distal base to the forefoot segment’s distal end (MTPJ joint center; blue circle) [37, 42]. We used the arc length equation to calculate the instantaneous arc length of the MTPJ as the product between the MTPJ angle (θ) and the radius of the metatarsal head (r) [36]. Finally, we calculated the total length of the total plantar structures as the sum of the arch length and the MTPJ wrapping length.

Participants walked barefoot for 5 minutes at 1.25 m s^-1^ on an instrumented split-belt treadmill (Bertec, Columbus OH, US) on level (0°), downhill (−10°, −5°), and uphill (+10°, +5°), in a randomized order within the same session (Fig 1 panel: A). In addition, participants wore tight-fitting ’wrestling’ suits. This suit promoted accurate and consistent placement of retro-reflective markers.

### Data collection: Kinematics and kinetics

Using a 14-camera motion capture system (Vicon, Oxford, UK), we collected lower extremity kinematics relative to the global reference system of the laboratory (100 Hz). At the beginning of the data collection session, we placed retro-reflective markers at specific anatomical bony landmarks of the feet, ankles, knees, and hips on each participant’s lower extremities [15, 47, 48], we secured them using double-sided tape, and we did not move the markers until the end of the data collection. Rigid mark clusters tracked the movements of the shank, thigh, and pelvis. The selection of the foot landmarks was adapted from a previously published multi-segment foot model by Bruening et al. (2012a, 2012b) and divided the foot into three segments: a) hindfoot (calcaneus and talus), (b) forefoot (navicular, cuboid, cuneiforms, and metatarsals), and (b) hallux (proximal and distal phalanges). We separated the hindfoot and the shank by the ankle joint, and the midtarsal joint separated the hindfoot and forefoot segments. We defined the midtarsal joint as the midpoint between the navicular and cuboid bone markers. The MTPJ separated the forefoot and hallux segments. The MTPJ joint center was estimated by projecting the first metatarsal head marker downward half the distance to the floor when the participant’s foot was flat on a level treadmill surface. We modeled the midtarsal, ankle, knee, and hip joints as six-degree-of-freedom joints. The MTPJ joint was modeled with two-degree-of-freedom permitting: dorsiflexion/plantarflexion and adduction/abduction [47].

Kinetic data were measured using an instrumented split-belt treadmill (Bertec, Columbus OH, USA) with a 1000 Hz sampling rate. To minimize the error in the center of pressure estimates, we used a rigid, rod-shaped testing device and the CalTester function in Visual3D (C-motion, Germantown, MD, USA) to assess the accuracy of the force platforms when used in conjunction with the motion capture system [49]. We found an average error between force platforms of x: 2.25±0.95, y: 0.91±0.76, and z: 0.06±0.75 mm for level (0°), x: -0.28±0.13, y: 1.49±0.59, and z: -0.11±0.11 mm for uphill (+5°) and x: 0.65±0.17, y: 0.91±0.08 and z: 0.25±0.05 mm for uphill (+10°) (x: mediolateral, y: anterior-posterior, z: inferior-superior; mean±s.d. of the two force platforms).

We filtered the raw data from marker trajectories and ground reaction forces by applying a second-order dual-pass low-pass Butterworth filter of 6 Hz for kinematic data and 25 Hz for kinetic data. A 20 N threshold for the vertical ground reaction force defined the start and the end time for each stance phase of walking.

### Analysis: Distal-to-hindfoot (i.e., structures of the entire foot) mechanical power and work

To estimate the mechanical power of the foot, we used a unified deformable (UD) segment analysis [41]. We chose the hindfoot segment as the reference coordinate system (rigid component) and modeled all the structures distal to the hindfoot as the distal deformable component. That analysis captures the summed mechanical contribution of heel pad deformation and all other foot structures, crossing the mid-tarsal and metatarsophalangeal joints and hallux segment [16]. This analysis has also been described as the power/work of the ’hindfoot relative to the ground’ [50].

In summary of our analysis, we quantified the total mechanical power of structures distal to the hindfoot *P*_*hf*_*dist*_, using the following equation:

$$P_{hf\_dist}={\vec{F}}_{gnd}∙{\vec{v}}_{hf\_dist}+({\vec{M}}_{free}∙{\vec{\omega }}_{hf})$$

where ${\vec{F}}_{gnd}$ is the ground reaction force, ${\vec{M}}_{free}$ is the free moment, ${\vec{\omega }}_{hf}$ is the angular of the hindfoot segment (based on a rigid body assumption) and ${\vec{v}}_{hf\_dist}$ is the translational velocity coincident with the center-of-pressure location, using the following equation:

$${\vec{v}}_{hf\_dist}={\vec{v}}_{hf\_com}+({\vec{\omega }}_{hf\_dist}\times {\vec{r}}_{COP/hf\_dist})$$

where ${\vec{v}}_{hf\_com}$ is the translational velocity of the center-of-mass of the hindfoot segment (based on a rigid body assumption) and ${\vec{r}}_{cop/hf\_dist}$ is the displacement of the center of pressure relative to the center of mass of the hindfoot. That analysis assumes that the contribution of inertial effects is zero [41] and does not account for the radial velocity component of the ${\vec{r}}_{cop/hf\_dist}$ vector since including this term can lead to a power imbalance in a foot segment [51].

### Analysis: Sub-dividing the distal-to-hindfoot mechanical power and work

We determined the timing when the COP was posterior to the midtarsal joint. Then, we partitioned the early phase of the distal-to-hindfoot power stance using methods we described in our previous work [15, 40]. Briefly, we compared the anterior-posterior positions of the COP relative to the midtarsal joint in the laboratory coordinate system, and we determined the timing in which the COP crosses the joint anteriorly. We expected that the work performed during the sub-phase (when the COP is posterior to the midtarsal joint) would include contributions from structures underneath the hindfoot (e.g., heel pad) and contributions from tissues crossing the midtarsal joint.

### Analysis: Midtarsal joint & distal-to-forefoot mechanical power and work

To further subdivide power contributions from the foot, we quantified the contributions of the midtarsal joints and structures distal to the forefoot’s center of mass [15, 16, 22, 52]. Previous research has shown that the summation of midtarsal joint and distal-to-forefoot powers was nearly equivalent to the total distal-to-hindfoot power [16]. Therefore, we have verified that the summation of midtarsal joint and distal-to-forefoot powers is nearly equal to distal-to-hindfoot power (S1 Fig).

We quantified the total power of the midtarsal joint using a six-degree-of-freedom model [17, 43] because such an approach improves the agreement between work produced by the lower extremity structures and the energy change of the body [17]. We assumed that the midtarsal joint moment and forces are zero before the center-of-pressure passing anterior to the joint center to quantify midtarsal joint power [40]. While such an approach possibly misses a portion of the midtarsal joint moment and negative work during early stance, previous research found that the positive work estimates from this method are accurate [40].

To quantify distal-to-forefoot power, we used a unified deformable segment analysis to model all the structures distal to the forefoot’s center of mass as a deforming segment [15, 16, 40]. Previous studies have described the mathematical approach to quantifying the distal-to-forefoot power using a unified deformable (UD) segment analysis [15, 16, 40]. The equations are identical to the distal-to-hindfoot power calculations (see above) but expressed relative to the forefoot segment. That analysis captures the summed mechanical contribution of structures surrounding the metatarsophalangeal joints and hallux segment [16]. We quantified distal-to-forefoot power and work only when the center of pressure was anterior to the midtarsal joint.

### Analysis: Ankle, knee, and hip joint mechanical power and work

We also quantified the total power of the ankle, knee, and hip joints using a six-degree-of-freedom model [17, 43].

**Fig 2:**
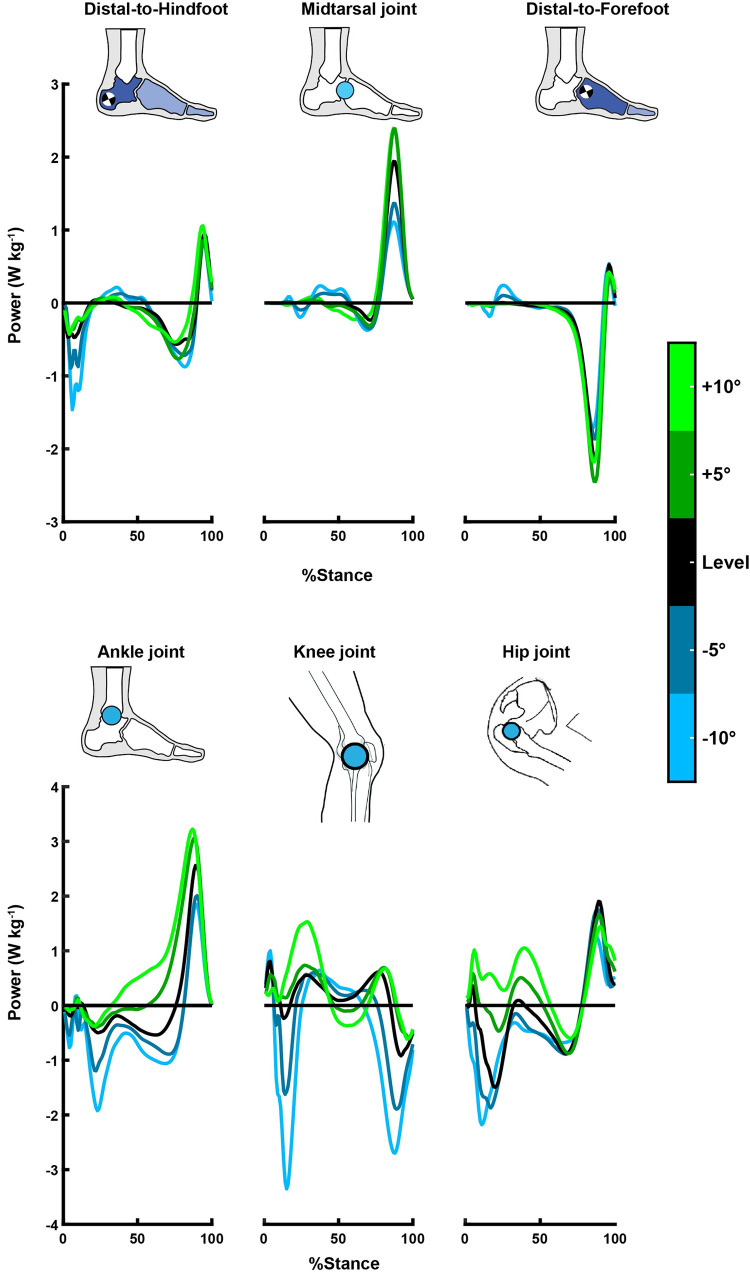
Average mechanical power over stance period for all the walking conditions: Level (0°), downhill (−5°,−10°), and uphill (+5°,+10°) (n = 12). **(Top)** Distal-to-hindfoot (i.e., entire foot), midtarsal joint, and distal-to-forefoot power timer series. **(Bottom)** Ankle, knee, and hip joints. The power time series are normalized to body mass. The y-axis limits differ between the power of the foot structures (top) and the rest of the leg’s joint powers (bottom).

Finally, for all of our mechanical power data, we quantified the negative and positive mechanical work over the entire stance phase by integrating the negative and positive portions of the mechanical power data over time. Net work was calculated as the summation of positive and negative work. We normalized the work values by participant’s biological mass because we used slope walking to determine the potential of the foot to perform work during nonzero net mechanical work locomotor tasks (Fig 2).

### Analysis: Kinematics of the plantar structures crossing the longitudinal arch and the MTPJ

We estimated the horizontal length of the longitudinal arch (i.e., arch length) by measuring the distance from the hindfoot’s distal base to the forefoot segment’s distal end (MTPJ joint center) [37, 42]. Then, we approximated the wrapping length of the plantar structures, like the plantar fascia, around the head of the first metatarsal, as a function of the MTPJ angle (Fig 1 panel: B). We did that by using the arc length equation to calculate the instantaneous arc length of the MTPJ as the product between the MTPJ angle and the radius of the metatarsal head [36]. To estimate the radius of the metatarsal head, we projected the midpoint between the first metatarsal head marker and the vertical projection on the floor. Finally, we calculated the total length of plantar structures as the sum of the horizontal length of the longitudinal arch (i.e., arch length) and the MTPJ wrapping length (Fig 1 panel: B).

We calculated the shortening displacement of the arch length as the difference between the length value during the heel strike and the corresponding minimum value during the toe-off phase (i.e., the late state of the push-off phase). We also calculated the lengthening displacement of the MTPJ wrapping length and the total length of plantar structures as the difference from the heel strike to the corresponding peak length value during the toe-off phase (i.e., the late state of the push-off phase). The MTPJ displacement was calculated as the difference from the heel strike to the peak dorsiflexion angle. We normalized the arch length, MTPJ wrapping length, and the total length of plantar structures by the participant’s foot length.

While our method primarily captures the kinematics of plantar fascia, we acknowledge that other plantar structures, like muscles, have similar anatomical attachments to the plantar fascia [42, 53–55] and could contribute to the length changes that our analysis estimates.

For all our analyses, we examined approximately ten stance phase data series for each participant, right leg, and each walking condition for all of our analyses. We obtained the data in the middle of the 5-minute walking trials. We performed the data processing and analysis using Vicon motion analysis software (Vicon Nexus, Oxford, UK), Visual3D (C-motion, Germantown, MD, USA), and MATLAB R2021a (MathWorks, Natick, MA, USA). We reported the values in the result section as mean±sd.

### Statistical analysis: Hypotheses testing

We checked all the dependent variables for normality, sphericity, and outliers. We used one-factor (three levels of slope) repeated-measures ANOVAs (IBM SPSS Statistics 26, SPSS Inc., Chicago, IL, USA) to compare level walking vs. downhill or uphill walking and determine the slope’s effect on the dependent variables (negative, positive, net mechanical work, arch and MTPJ displacement and peak dorsiflexion). We used a Bonferroni post hoc analysis to conduct the pair-wise comparisons test when we found a significant main effect. We used Friedman’s test if the assumptions of normality etc., were not met, correcting for pair-wise multiple comparisons using a Bonferroni post hoc analysis. For all our statistical analyses, we set the significance level to α = 0.05.

## Results

**Fig 3:**
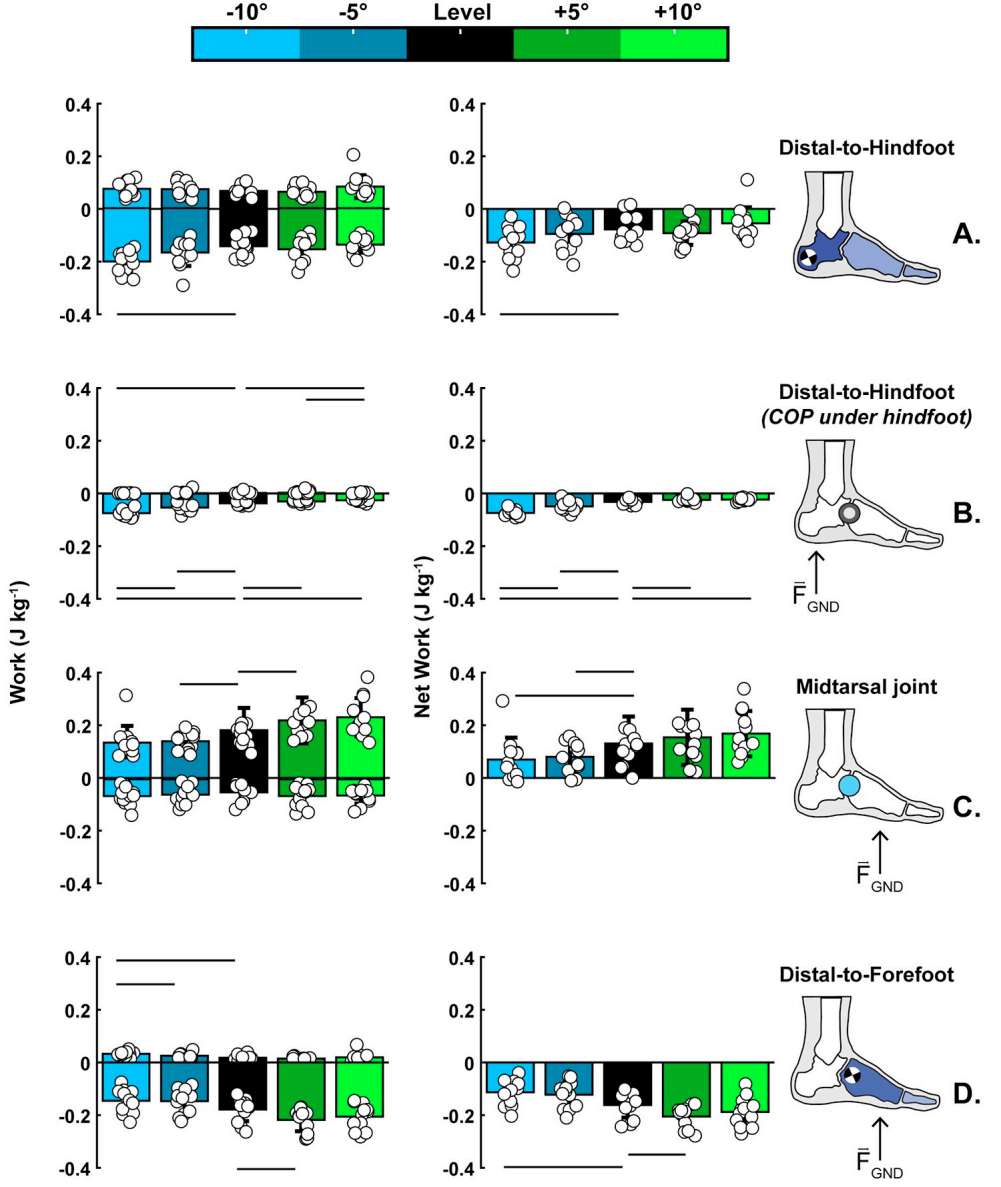
Average negative, positive, and net work data for all the walking conditions: Level (0°), downhill ((−5°,−10°), and uphill (+5°,10°) (n = 12). We compared level walking with downhill or uphill walking, and the horizontal straight lines indicated the pairwise significance between walking conditions. **(Α)** The foot (i.e., distal-to-hindfoot) maintained its net negative work behavior across all walking conditions, with only downhill walking increasing the magnitude of the foot’s negative (p<0.001) and net negative (p = 0.013) work. **(B)** When the center of pressure was under the hindfoot, the magnitude of the net negative work increased during downhill walking (0° to −5°: p = 0.016; 0° to −10°: p<0.001; −5° to −10°: p<0.001) and decreased during uphill walking (0° to +5°: p = 0.002; 0° to +10°: p = 0.012). **(C)** The midtarsal joint produced net positive work across all walking conditions, with downhill walking decreasing the net positive work (0° to −5°: p = 0.002; 0° to −10°: p = 0.023). **(D)** The structures distal-to-forefoot produced net negative work across all waking conditions, with downhill walking decreasing the net negative work (0° to −10°: p = 0.020) and uphill walking increasing it (0° to +5°: p = 0.005). The work values are normalized to body weight. The rest of the p-values can be found in the text of the result section.

**Fig 4:**
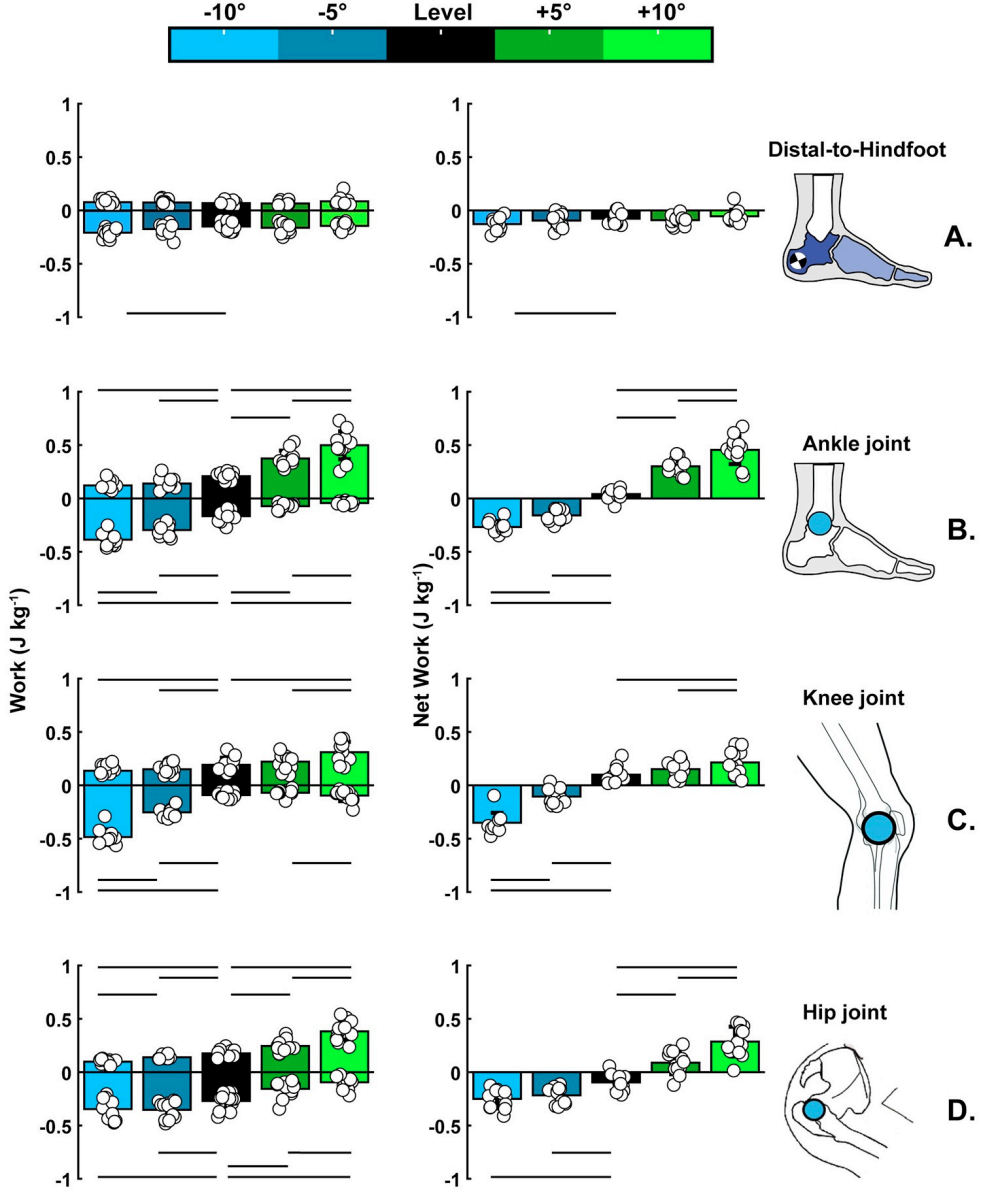
Average negative, positive, and net work data for all the walking conditions: Level (0°), downhill (−5°,−10°), and uphill (+5°,+10°) (n = 12). We compared level walking with downhill or uphill walking, and the horizontal straight lines indicated the pairwise significance between walking conditions. **(A)** The foot (i.e., distal-to-hindfoot) maintained its net negative work behavior across all walking conditions, with only downhill walking increasing the magnitude of the foot’s negative (p<0.001) and net negative (p = 0.013) work. **(B)** Downhill walking shifted and increased the ankle’s net mechanical behavior (0° vs. −5°: p<0.001; 0° vs −10°: p<0.001; −5° vs. −10°: p<0.001). During uphill walking, the ankle increased the magnitude of the net positive work when the slope of walking increased (0° vs. +5°:p = 0.002; 0° vs. +10°: p = 0.002; +5° vs. +10° p = 0.002). **(C)** Downhill walking shifted and increased the knee’s net mechanical behavior (0° vs. −5°: p = 0.002; 0° vs −10°: p = 0.002; −5° vs. −10°: p = 0.002). During uphill walking, the knee increased the magnitude of net positive work (0° vs. 10°: p = 0.005; +5° vs. +10°: p = 0.012). **(D)** The hip increased the magnitude of the net negative work when the slope of walking decreased (0° vs. −5°: p = 0.002; −0° vs. −10°: p = 0.002), while uphill walking shifted and increased the hip’s net mechanical behavior (0° vs. +5°: p = 0.002; 0° vs +10°: p = 0.002; +5° vs. +10°: p = 0.002). The work values are normalized to body weight. The rest of the p-values can be found in the text of the result section.

In summary, the foot maintained its net negative work across all walking conditions, with only downhill walking increasing the magnitude of the foot’s negative (p<0.001) and net negative (p = 0.013) work (Figs 3 and 4, panel: A). However, at the ankle, knee, and hip joints, sloped walking altered the net mechanical work behavior from net negative during downhill walking to net positive during uphill walking (Fig 4, panels: B-D).

Additionally, when the center of pressure was under the hindfoot (posterior to the midtarsal joint), the magnitude of the net negative work significantly increased (p<0.001) during downhill and decreased during uphill walking (p = 0.006; Fig 3, panel: B). The midtarsal joint produced net positive work across all walking conditions, with downhill and uphill walking decreasing and increasing (respectively) the positive work significantly (p = 0.009 & p = 0.017; respectively; Fig 3, panel: C). Interestingly, the structures distal-to-forefoot decreased the magnitude of the net negative work during downhill and increased it during uphill walking (p = 0.011 & p = 0.005, respectively; Fig 3, panel: D).

**Fig 5:**
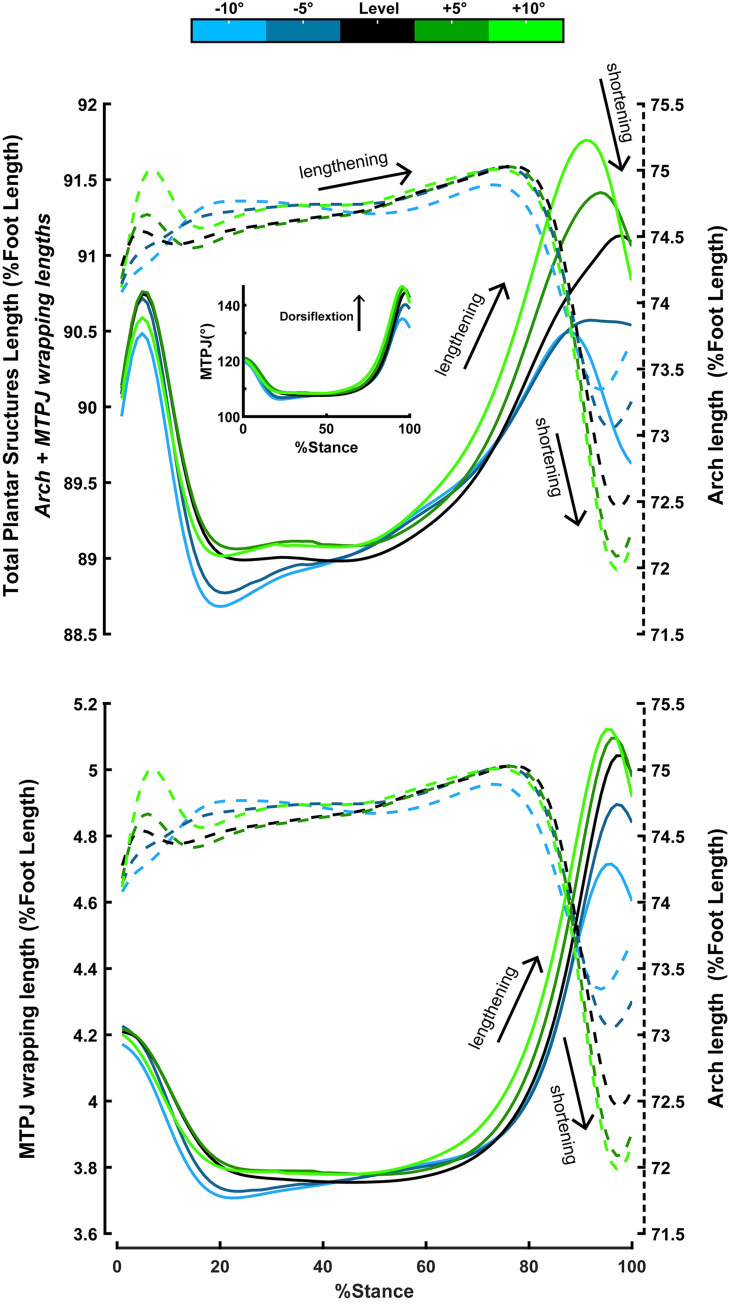
Average kinematics time series, normalized to the foot length (% foot length) over stance period for all the walking conditions: Level (0°), downhill (−5°,−10°), and uphill (+5°,+10°) (n = 12). **(Top)** On the left y-axis, the average of the total length of the plantar structures is calculated as the sum of the arch and the MTPJ wrapping lengths (i.e., arch + MTPJ wrap lengths). On the right, the dashed y-axis displays the arch length. In the middle is displayed the average MTPJ angle. **(Bottom)** On the left y-axis is the average MTPJ wrapping length, and on the right dashed y-axis is the arch length. Downhill walking decreased the peak MTPJ dorsiflexion angle (0° to −10°: p<0.001; 0° to −5°: p<0.001; −5° to −10°: p<0.001), the displacement of the MTPJ wrapping (0° to −10°: p<0.001; 0° to −5°: p = 0.001; -5° to −10°: p = 0.012), the arch length displacement (0° to −10°: p<0.001; 0° to −5°: p<0.001; -5° to −10°: p = 0.004), and the displacement of the total length of the plantar structures (0° to −10°: p = 0.002; 0° to −5°: p = 0.001). In contrast, uphill walking increased the peak MTPJ dorsiflexion angle (0° to +5°: p = 0.023; 0° to +10°: p = 0.023), the displacement of the MTPJ wrapping length (0° to +10°: p = 0.041), the displacement of the arch length (0° to +5°: p = 0.020), and the displacement of the total length of the plantar structures (0° to +10°: p = 0.040).

Finally, downhill walking decreased while uphill walking increased the peak MTPJ dorsiflexion angle (Fig 5; p<0.001 & p = 0.009, respectively). Also, downhill walking decreased the shortening displacement of the arch length (p<0.001), the lengthening displacement of the MTPJ wrapping length (p<0.001), and the total lengthening of the plantar structures (i.e., arch + MTPJ wrap lengths) (p<0.001), while uphill walking increased their displacement (arch length: p = 0.024; MTPJ wrapping length: p = 0.005; total length: p = 0.018).

### Distal-to-hindfoot work during stance

Downhill walking had a statistically significant effect on the negative (p<0.001) and net work (p = 0.013) but not on the positive work (p = 0.779). The foot increased the magnitude of the negative work significantly when the slope of walking decreased from 0° to −10° (p<0.001; -0.146±0.039 vs. -0.204±0.041 Jkg^-1^; respectively). The magnitude of net negative work significantly increased when the slope of walking decreased from 0° to −10° (p = 0.016; -0.078±0.054 vs. -0.128±0.062 Jkg^-1^; respectively).

Uphill walking had no statistically significant effect on the magnitude of the foot’s negative (p = 0.197), positive (p = 0.108), or net work (p = 0.057).

### Work when the center of pressure is under the hindfoot

When the center of pressure was underneath the hindfoot, downhill walking had a statistically significant effect on the negative (p<0.001), positive (p = 0.017), and net work (p<0.001) of the foot. The magnitude of the negative work significantly increased when the slope of walking decreased from 0° to −10° (p<0.001; -0.037±0.007 vs. -0.075±0.014 Jkg^-1^; respectively), from 0° to −5° (p = 0.005; -0.037±0.007 vs. -0.053±0.015 Jkg^-1^; respectively) and from −5° to −10° (p<0.001; -0.053±0.015 vs. -0.075±0.014 Jkg^-1^; respectively). The magnitude of the positive work significantly decreased when the slope of walking decreased from 0° to −10° (p<0.001; 0.004±0.004 vs. 0.001±0.001 Jkg^-1^; respectively). The magnitude of the net negative work significantly increased when the slope of walking decreased from 0° to −10° (p<0.001; -0.032±0.009 vs. -0.074±0.014 Jkg^-1^; respectively), from 0° to −5° (p = 0.016; -0.032±0.009 vs. -0.049±0.019 Jkg^-1^; respectively) and from −5° to −10° (p<0.001; -0.049±0.019 vs. -0.074±0.014 Jkg^-1^; respectively).

Uphill walking had a statistically significant effect on the negative (p<0.001), positive (p = 0.005), and net work (p = 0.006). The magnitude of the negative work significantly decreased when the slope of walking increased from 0° to +10° (p = 0.002; -0.037±0.007 vs. -0.025±0.006 Jkg^-1^; respectively), from 0° to +5° (p = 0.004; -0.037±0.007 vs. -0.030±0.005 Jkg^-1^; respectively). The magnitude of the positive work significantly decreased when the slope of walking increased from 0° to +10° (p = 0.010; 0.004±0.004 vs. 0.002±0.002 Jkg^-1^; respectively) and from +5° to +10° (p = 0.002; 0.005±0.006 vs. 0.002±0.002 Jkg^-1^; respectively). The magnitude of the net negative work significantly decreased when the slope of walking increased from 0° to +10° (p = 0.012; -0.032±0.009 vs. -0.023±0.006 Jkg^-1^; respectively) and from 0° to +5° (p = 0.002; -0.032±0.009 vs. -0.025±0.008 Jkg^-1^; respectively).

### Midtarsal work during stance

Downhill walking had a significant effect on midtarsal joint positive (p = 0.009) and net (p<0.001) work but not on negative work (p = 0.059) of the midtarsal joint. The magnitude of the positive work significantly decreased when the slope of walking decreased from 0° to −5° (p = 0.005; 0.181±0.085 vs. 0.139±0.062 Jkg^-1^; respectively). The magnitude of the net positive work significantly decreased when the slope decreased from 0° to −10° (p = 0.023; 0.131±0.102 vs. 0.070±0.084 Jkg^-1^; respectively) and from 0° to −5° (p = 0.002; 0.131±0.102 vs. 0.080±0.058 Jkg^-1^; respectively).

Uphill walking had a significant effect on positive (p = 0.017) but not on the negative (p = 0.100) or net (p = 0.078) work at the midtarsal joint. The magnitude of the positive work significantly increased when the slope of walking increased from 0° to +5° (p = 0.012; 0.181±0.085 vs. 0.219±0.087 Jkg^-1^; respectively).

### Distal-to-forefoot work during stance

Downhill walking had a significant effect on positive (p = 0.002) and net (p = 0.011) but not on negative (p = 0.069) work. The magnitude of positive work significantly increased when the slope of walking decreased from 0° to −10° (p = 0.002; 0.018±0.010 vs. 0.033±0.008 Jkg^-1^; respectively) and from −5° to −10° (p = 0.029; 0.026±0.009 vs. 0.033±0.008 Jkg^-1^; respectively). The magnitude of net negative work significantly decreased when the slope of walking decreased from 0° to −10° (p = 0.020; -0.162±0.048 vs. -0.113±0.048 Jkg^-1^; respectively).

Uphill walking had a significant effect on the negative (p = 0.005) and net (p = 0.005) but not on the positive(p = 0.558) work. The magnitude of the negative work significantly increased when the slope of walking increased from 0° to +5° (p = 0.003; -0.180±0.045 vs. -0.220±0.044 Jkg^-1^; respectively). The magnitude of the net negative work significantly increased when the slope of walking increased from 0° to +5° (p = 0.005; -0.162±0.048 vs. -0.205±0.042 Jkg^-1^; respectively).

### Kinematics of the plantar structures crossing the longitudinal arch and the MTPJ during stance

Downhill walking had a significant effect on the shortening displacement (%foot length) of the arch (p<0.001), the lengthening displacement of the MTPJ wrap (p<0.001), and the total lengthening of the plantar structures (i.e., arch + MTPJ wrap lengths) (p<0.001), as well as the peak MTPJ dorsiflexion angle (p<0.001). The peak MTPJ dorsiflexion angle decreased when the slope decreased from 0° to −10°(p<0.001; 146±7.40 vs. 137±7.50 (°); respectively), from 0° to −5° (p<0.001; 146±7.40 vs. 142±7.23 (°); respectively), and from −5° to −10° (p<0.001; 142±7.23 vs. 137±7.50 (°); respectively). The MTPJ wrapping lengthening decreased when the slope decreased from 0° to −10°(p<0.001; 25.34±5.14 vs.17.0±5.29 (°); respectively), from 0° to −5° (p = 0.001; 25.34±5.14 vs. 21.0±4.16 (°); respectively), and from -5° to −10° (p = 0.012; 21.0±4.16 vs. 17.0±5.29 (°); respectively). The arch shortening displacement decreased when the slope decreased from 0° to −10°(p<0.001), from 0° to −5° (p<0.001), and from -5° to −10° (p = 0.004). The total lengthening of plantar structures (i.e., arch + MTPJ wrap lengths) decreased when the slope decreased from 0° to −10° (p = 0.002) and from 0° to −5° (p = 0.001).

Uphill walking had a significant effect on the shortening displacement (%foot length) of the arch (p = 0.024), the lengthening displacement of the MTPJ wrap (p = 0.005), the total lengthening of the plantar structures (i.e., arch + MTPJ wrap lengths) (p = 0.018), as well as the peak of the MTPJ dorsiflexion angle (p = 0.009). The peak MTPJ dorsiflexion angle increased from 0° to +5° (p = 0.023) and from 0° to +10° (p = 0.023). The total lengthening of the plantar structures increased when the slope increased from 0° to +10° (p = 0.040). The shortening displacement of the arch increased when the slope increased from 0° to +5° (p = 0.020). The lengthening of the MTPJ wrap increased from 0° to +10° (p = 0.041).

### Ankle work during stance

Downhill walking had a significant effect on the negative (p<0.001), positive (p<0.001), and net (p<0.001) work at the ankle joint. The magnitude of the negative work significantly increased when the slope of walking decreased from 0° to −10° (p<0.001; -0.167±0.052 vs. -0.389±0.060 Jkg^-1^; respectively), from 0° to −5° (p<0.001; -0.167±0.052 vs. -0.299±0.053 Jkg^-1^; respectively) and from −5° to −10° (p<0.001; -0.299±0.053 vs. -0.389±0.060 Jkg^-1^; respectively). The magnitude of the positive work significantly decreased when the slope decreased from 0° to −10° (p<0.001; 0.209±0.033 vs. 0.122±0.040 Jkg^-1^; respectively) and from 0° to −5° (p<0.001; 0.209±0.033 vs. 0.140±0.052 Jkg^-1^; respectively). The net work altered from net positive to net negative when the slope of walking decreased from 0° to −10° (p<0.001; 0.042±0.047 vs. -0.267±0.056 Jkg-1; respectively), from 0° to −5° (p<0.001; 0.042±0.047 vs. -0.158±0.056 Jkg^-1^; respectively), and its magnitude increased from −5° to −10° (p<0.001; -0.158±0.056 vs. -0.267±0.056 Jkg^-1^; respectively).

Uphill walking had a significant effect on the negative (p<0.001), positive (p<0.001), and net (p<0.001) work at the ankle joint. The magnitude of the negative work significantly decreased when the slope of walking increased from 0° to +10° (p = 0.002; -0.167±0.052 vs. -0.044±0.014 Jkg^-1^; respectively), from 0° to +5° (p = 0.002; -0.167±0.052 vs. -0.073±0.025 Jkg^-1^; respectively) and from +5° to +10° (p = 0.002; -0.073±0.025 vs. -0.044±0.014 Jkg^-1^; respectively). The magnitude of the positive work significantly increased when the slope of walking increased from 0° to +10° (p = 0.002; 0.209±0.033 vs. 0.499±0.130 Jkg^-1^; respectively), from 0° to +5° (p = 0.002; 0.209±0.033 vs. 0.374±0.074 Jkg^-1^; respectively) and from +5° to +10° (p = 0.004; 0.374±0.074 vs. 0.499±0.130 Jkg^-1^; respectively). The magnitude of the net positive work significantly increased when the slope of walking increased from 0° to +10° (p = 0.002; 0.042±0.047 vs. 0.456±0.123 Jkg^-1^; respectively), from 0° to +5° (p = 0.002; 0.042±0.047 vs. 0.301±0.073 Jkg^-1^; respectively) and from +5° to +10° (p = 0.002; 0.301±0.073 vs. 0.456±0.123 Jkg^-1^; respectively).

### Knee work during stance

Downhill walking had a significant effect on the negative (p<0.001), positive (p = 0.001), and net (p<0.001) work at the knee joint. The magnitude of the negative work significantly increased when the slope of walking decreased from 0° to −10° (p = 0.002; -0.092±0.028 vs. -0.488±0.712 Jkg^-1^; respectively), from 0° to −5° (p = 0.002; -0.092±0.028 vs. -0.256±0.044 Jkg^-1^; respectively), and from −5° to −10° (p = 0.002; -0.256±0.044 vs. -0.488±0.712 Jkg^-1^; respectively). The magnitude of the positive work significantly decreased when the slope of walking decreased from 0° to −10° (p = 0.006; 0.192±0.066 vs. 0.137±0.056 Jkg^-1^; respectively), from 0° to −5° (p = 0.003; 0.192±0.066 vs. 0.150±0.047 Jkg^-1^; respectively). The net work altered from net positive to net negative 0° to −10° (p = 0.002; 0.100±0.073 vs. -0.351±0.093 Jkg-1; respectively), from 0° to −5° (p = 0.002; 0.100±0.073 vs. -0.105±0.740 Jkg^-1^; respectively), and its magnitude increased from −5° to −10° (p = 0.002; -0.105±0.740 vs. -0.351±0.093 Jkg^-1^; respectively).

Uphill walking had a significant effect on the negative (p = 0.046), positive (p = 0.001), and net (p = 0.005) work at the knee joint. The magnitude of negative work increased when the slope of walking increased from +5° to +10° (p = 0.012; -0.070±0.030 vs. -0.096±0.052 Jkg^-1^; respectively). The magnitude of positive work increased when the slope of walking increased from 0° to +10° (p = 0.001; 0.192±0.075 vs. 0.311±0.097 Jkg^-1^; respectively) and from +5° to +10° (p<0.001; 0.221±0.075 vs. 0.311±0.097 Jkg^-1^; respectively). The magnitude of net positive work increased when the slope of walking increased from 0° to +10° (p = 0.005; 0.100±0.073 vs. 0.214±0.117 Jkg^-1^; respectively) and from +5° to +10° (p = 0.012; 0.151±0.074 vs. 0.214±0.117 Jkg^-1^; respectively).

### Hip work during stance

Downhill walking had a significant effect on the negative (p = 0.004), positive (p<0.001), and net (p<0.001) work at the hip joint. The magnitude of the negative work significantly increased when the slope of walking decreased from 0° to −5° (p = 0.002; -0.273±0.079 vs. -0.356±0.082 Jkg-1; respectively) and from −0° to −10° (p = 0.002; -0.273±0.079 vs. -0.349±0.097 Jkg-1; respectively). The magnitude of the positive work significantly decreased when the slope of walking decreased from 0° to −10° (p = 0.002; 0.176±0.033 vs. 0.098±0.023 Jkg-1; respectively), from 0° to −5° (p = 0.002; 0.176±0.033 vs. 0.139±0.018 Jkg-1; respectively), and from −5° to −10° (p = 0.002; 0.139±0.018 vs. 0.098±0.023 Jkg-1; respectively). The magnitude of the net negative work significantly increased when the slope of walking decreased from 0° to −5° (p = 0.002; -0.097±0.077 vs. -0.217±0.077 Jkg-1; respectively) and from 0° to −10° (p = 0.002; -0.097±0.077 vs. -0.250±0.089 Jkg-1; respectively).

Uphill walking had a significant effect on the negative (p<0.001), positive (p<0.002), and net (p<0.001) work at the hip joint. The magnitude of the negative work significantly decreased when the slope of walking increased from 0° to +10° (p = 0.002; -0.273±0.079 vs. -0.096±0.061 Jkg^-1^; respectively), from 0° to +5° (p = 0.002; -0.273±0.079 vs. -0.158±0.079 Jkg^-1^; respectively), and from +5° to +10° (p = 0.002; -0.158±0.079 vs. -0.096±0.061 Jkg^-1^; respectively). The magnitude of the positive work significantly increased when the slope of walking increased from 0° to +10° (p = 0.002; 0.176±0.033 vs. 0.383±0.094 Jkg^-1^; respectively), from 0° to +5° (p = 0.002; 0.176±0.033 vs. 0.245±0.055 Jkg^-1^; respectively), and from +5° to +10° (p = 0.002; 0.245±0.055 vs. 0.383±0.094 Jkg^-1^; respectively). The magnitude of net positive work significantly increased between +5° to +10° (p = 0.002; 0.088±0.108 vs. 0.287±0.140 Jkg^-1^; respectively), while altered from net negative to net positive when the slope of walking increased from 0° to +10° (p = 0.002; -0.097±0.076 vs. 0.287±0.140 Jkg^-1^; respectively), from 0° to +5° (p = 0.002; -0.097±0.077 vs. 0.088±0.108 Jkg^-1^; respectively).

## Discussion

When faced with locomotion on sloped surfaces, animals, including humans, modulate the absorption, dissipation, and generation of mechanical work of the leg’s joints [1–7, 18, 20, 21]. Notably, the current study quantified how the various subsections of the human foot modulate their mechanical work and power production with respect to the slope of walking. We have shown that the human foot maintains its net negative mechanical work behavior between uphill and downhill slopes, with only downhill walking increasing the magnitude of the foot’s negative (p<0.001) and net negative (p = 0.013) work (Figs 3 and 4 panel: A). In contrast, sloped walking shifted the net mechanical work behavior of the ankle, knee, and hip joints from net positive to net negative when the walking surface changed from uphill to downhill (Fig 4 panels: B-D). These results broaden our knowledge about the mechanical function of the foot and leg joints during daily locomotor tasks.

More specifically, our data confirmed our hypotheses about downhill walking. We hypothesized that compared to level walking, downhill walking would increase the foot’s negative and net negative work during the entire and during the early stance phase highlighting the foot’s role as a shock absorber. When the slope of walking decreased from 0° to −10°, the magnitude of the foot’s negative work increased by 40% (p<0.001) and net negative work by 65% (p = 0.016) (Figs 3 and 4, panel: A). Notably, during the early stance phase, the magnitude of the negative work increased by 104% (p<0.001) and net negative work by 129% (p<0.001) between 0° to −10° (Fig 3, panel: B). The magnitude of net negative work during the early stance phase of level walking (-0.032 Jkg^-1^ or -2.357 J) was fairly similar to previous walking studies [15, 39, 56]. During the early stance phase of downhill walking on a −10° slope, that dissipative behavior by the foot increased substantially (-0.074 Jkg^-1^ or -5.514 J). While the tissues crossing the midtarsal joint contributed to some of that negative work [40, 42], such extensive energy damping requirements surpassed the suggested energy levels (≈2.12 J and ≈3.7 J) of the heel’s pain tolerance during deformation [57–59] and possibly increased the risk of injuries [60, 61]. However, none of our participants complained about pain or reported any injury. Therefore, these findings raise intriguing questions regarding how this energy is utilized by the body or dissipated by the foot and if excessive energy dissipation is associated with tissue mechanical properties like thickness and stiffness and contributes to foot complications like plantar heel pain [62]. Nevertheless, our study showed that downhill walking exaggerated the dissipative behavior of the healthy foot during the entire and early phase of each step, suggesting that the foot contributes to the dissipation of impacts by the legs during downhill walking [3].

For uphill walking, our data partially support our hypotheses. We hypothesized that compared to level walking, uphill walking would increase the positive work performed by the foot (i.e., distal-to-hindfoot) during the entire stance and particularly at the mid-to-late stance phase. However, uphill walking had no statistically significant effect on the magnitude of the foot’s positive work (p = 0.108), rejecting the first part of our hypothesis (Figs 3 and 4 panel: A). Yet, during the mid-to-late stance phase, the structures crossing the midtarsal joint increased their positive work when the slope of walking increased from 0° to +5° (p = 0.012; 0.181±0.085 vs. 0.219±0.087 Jkg^-1^; respectively), partially confirming the last part of our hypothesis. (Fig 3, panel: C). Nevertheless, that positive work production from the midtarsal joint coincided with a nearly equal increase in the magnitude of negative work by the structures distal to the forefoot (p = 0.003; 0°: -0.180±0.045 vs. +5°:-0.220±0.044 Jkg^-1^; Fig 3, panel: D). The nearly equal amounts of positive and negative work during the mid-to-late stance possibly explain why the distal-to-hindfoot analysis (i.e., structures of the entire foot) did not reveal a statistical increase in the foot’s positive work (Figs 3 and 4 panel: A). However, such opposing work production from the midtarsal joint and the distal-to-forefoot structures during the mid-to-late stance phase of uphill walking may result from the elastic function of the plantar structures that cross the longitudinal arch and wrap around the MTPJ (Fig 5).

The dorsiflexion of the MTPJ is one way to engage and enhance the elastic function of the longitudinal arch’s plantar structures [33, 37]. Our results showed that uphill walking increased the MTPJ dorsiflexion angle (p = 0.009) (Fig 5: Top-middle). That increase prolonged the lengthening (i.e., stretching) of the total length of the plantar structures that cross the arch and MTPJ and delayed their shortening (i.e., recoil) further in the late stance phase (Fig 5: Top; left y-axis). If we assume that our geometric model describes only the spring-like behavior of passive-elastic structures, like the plantar fascia, the prolonged and increased lengthening during uphill walking possibly allowed the plantar fascia to store more negative work and return it during the late stance. Therefore that may allow the plantar fascia to operate nearly elastically by storing negative work at distal structures of the forefoot like the metatarsal-phalangeal joints [35, 63] and returning positive work at the metatarsal joint at the late period of push-off [35] (Fig 3). However, our geometric model analysis cannot rule out the contribution of the foot’s intrinsic muscles. Plantar intrinsic foot muscles have similar anatomical attachments to the plantar fascia [42, 53–55] and, despite their small size, contribute to the mechanical demands of locomotor tasks [22, 30, 31, 42]. Previous studies showed that plantar foot muscles lengthen during the first half of the stance, and they shorten while the arch length shortens during the mid-to-late stance [42, 64]. However, our geometric model cannot precisely identify whether passive-elastic structures or active muscles that cross the midtarsal and MTP joint are responsible for our observed mechanical work production. Nevertheless, our joint/segment-based results showed that the foot subsections produce nearly equal amounts of positive and negative work during the mid-to-late stance (Fig 3), which increased during uphill walking. Such mechanical behavior possibly indicates the foot’s capacity to assist with the increased propulsion demands of the task [30]. Further experiments must explore the contribution of plantar passive-elastic structures and active muscles to the foot’s elastic behavior and stiffness during daily locomotor activities.

In conclusion, our results indicate that the foot exaggerates its dissipative behavior during locomotion that demands excessive energy dissipation, like downhill walking, highlighting its role as an impact absorber (Fig 3 panel: B). Notably, the foot maintained its overall dissipative behavior even across walking tasks that required a substantial increase in the body’s net-positive mechanical work, like uphill walking (Fig 3 panel: A). However, in contrast to the foot and its subsections, the ankle, knee, and hip joints shifted their net mechanical behavior between uphill and downhill walking, generating, and dissipating more energy than the foot (Fig 4 panels: B-D). Therefore, our results indicate that humans rely more on joints proximal to the foot to modulate the body’s total mechanical energy. Also, distal structures of the foot, like the midtarsal joint and the distal-to-forefoot structures, perform nearly equal amounts of positive and negative work during the mid-to-late stance, possibly highlighting the elastic function of the plantar structures of the foot, as previous studies suggest [32, 33, 35–37, 42, 65]. The kinematic results of the plantar structures suggest that the dorsiflexion of the MTPJ prolonged and increased their lengthening and delayed their shortening during uphill walking, possibly enhancing their elastic function by allowing them to store more negative work and return it during the late stance. Overall, our results expand the knowledge of foot energetics, highlighting the dissipation of energy as its primary function during walking tasks. Also, our results pointed out that the subjections of the foot can modulate their work to assist with the task’s increased propulsion demands when necessary. Such knowledge could inspire the design of footwear, prosthetics, and exoskeletons to restore and assist users’ walking capacity. A natural progression of this work is to analyze to which extent active and passive plantar and dorsal structures drive the net dissipative behavior of the foot.

## Supporting information

S1 Fig Verifying that the summation of midtarsal joint and distal-to-forefoot powers is nearly equal to the distal-to-hindfoot power. Summation of midtarsal joint and distal-to-forefoot powers (black line) and distal-to-hindfoot power (red line) over stance period for all the walking conditions: level (0°), downhill (−5°,−10°), and uphill (+5°,+10°) and for all the participants (each subplot represents the data for a participant).
(TIF)

S1 File (ZIP)
