# Supplementary figures and images for: Mechanics of the human foot during walking on different slopes
Source: PLoS One. 2023 Sep 11;18(9):e0286521. doi: 10.1371/journal.pone.0286521 (PMC10495022; doi:10.1371/journal.pone.0286521)

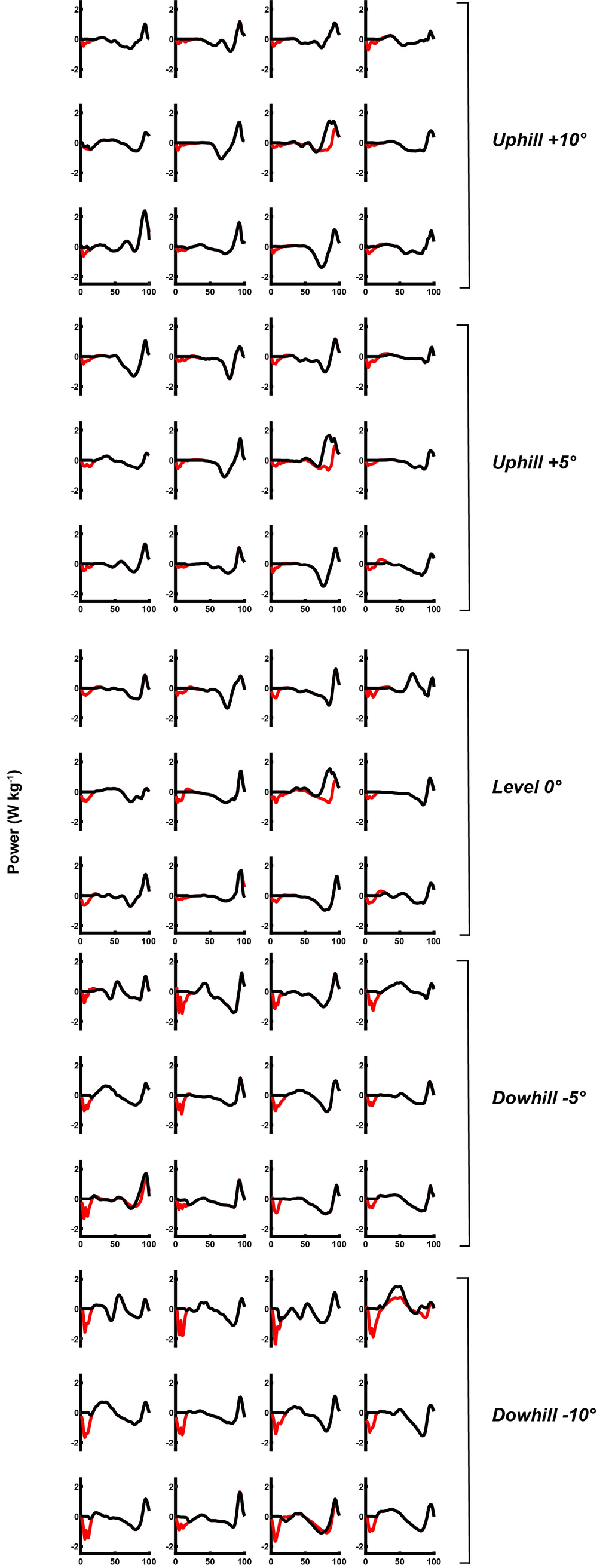

Supplement: S1 Fig — Summation of midtarsal joint and distal-to-forefoot powers (black line) and distal-to-hindfoot power (red line) over stance period for all the walking conditions: level (0°), downhill (−5°,−10°), and uphill (+5°,+10°) and for all the participants (each subplot represents the data for a participant). (TIF) [file pone.0286521.s001.tif]
